# Stable or changing well-being? Daily hassles and life satisfaction of Czech adolescents over the last three decades

**DOI:** 10.3389/fpsyg.2022.961373

**Published:** 2022-07-25

**Authors:** Petr Macek, Stanislav Ježek, Lenka Lacinová

**Affiliations:** Institute for Research on Children, Youth, and Family, Faculty of Social Studies, Masaryk University, Brno, Czechia

**Keywords:** well-being, daily hassles, life satisfaction, self-esteem, adolescence, social change

## Abstract

While the assumption that the sociopolitical and economic situation affects adolescents’ well-being, encompassing life satisfaction and a positive sense of self, is plausible, few studies have confirmed such macrosocial influences. The case of the Czech Republic offers an example of a society transitioning from totalitarian government (from 1989) to western democracy. Our study provides statistical description of Czech adolescents’ well-being over the past 30 years in association with the subjective perception of everyday problems. These daily hassles represent experiences and conditions of daily living that have been appraised as salient and harmful or threatening to adolescents’ well-being. We analyzed four samples of adolescents aged 14–17 years surveyed at four time points over the last three decades—1992, 2001, 2011, and 2019, total *N* = 4,005 (1992: 255, 2001: 306, 2011: 363, 2019: 3081; 54.6% females). The results show that life satisfaction, self-esteem, and self-reported daily hassles changed only marginally from 1992 to 2019 with small differences related to the post-revolution 1992 cohort. Adolescents reported increasing problems in school, relationships with parents, sports, and leisure time over the study period. A model linking daily hassles and self-esteem to life satisfaction across four cohorts showed that daily hassles strongly predicted life satisfaction except in the post-revolution cohort of 1992 when life satisfaction was also the lowest. The effect was slightly higher in females. Across the cohorts, gender differences in life satisfaction changed from males being more satisfied in 1992 to females being more satisfaction in 2019. Limitations stemming from sampling differences across cohorts are discussed.

## Introduction

In recent decades, a growing body of research has focused on adolescent well-being in non-clinical adolescent populations (Pollard and Lee, 2003; Ben-Arieh et al., 2014). Research that focuses primarily on physical and mental health often relies on the WHO definition of well-being, which emphasizes how people develop their own potential, their relationships, and their ability to cope with everyday stress (The World Health Organisation, 2013). In our research, we focus only on subjective well-being (SWB), which refers to an individual’s assessment of life (Moksnes and Espnes, 2013).

Widely accepted theoretical conceptualization of SWB (Diener, 1984, 2013) comprises three relatively independent components: perceived life satisfaction (cognitive component), positive experiences, and negative experiences (affective component). If more traditional approaches emphasize the presence or absence of negative feelings and experienced problems, then contemporary positive psychology posits that SWB is based more on positive quality of life indicators such as life satisfaction, happiness, self-esteem, and experiencing positive emotions.

From a developmental point of view, the content of SWB is not changing much, but the range of psychological phenomena influencing SWB expands with age. These include internal and external factors, including macrosocial determinants (social adaptation, cultural, social, and political factories). It also appears that the strength of some of the traditional personal and micro-social components or indicators of SBW is changing along with changing social conditions (Diener, 2013). However, capturing the direct effect of macrosocial changes is complicated. Objective indicators of quality of life, such as improved health care and more work or study opportunities, do not necessarily lead to higher SWB. People may find it difficult to adapt to changing life circumstances, and higher living standards may also increase personal aspirations, with new opportunities bringing new uncertainties (Diener et al., 2006). Moreover, in the adolescent population, the interplay of all the factors may be quite different from adult populations (Eccles et al., 2008).

Therefore, when studying the effects of social change on adolescents’ SWB, we need to consider both short-term and long-term effects. Cohort comparisons of samples gathered at different historical points are suitable designs to achieve this aim (Bronfenbrenner, 1979; Crockett and Silbereisen, 1999; Due et al., 2019). In the present cohort-comparison study, we focused on the extent to which personality characteristics (self-esteem), proximal situational influences (daily hassles), and changes in the macrosocial environment during the period of three decades (tracking four cohorts of adolescents at intervals of 8–9 years) influenced adolescents’ life satisfaction.

### Life satisfaction

Life satisfaction (LS) represents the cognitive component, a conscious and global evaluation of one’s own life, which can be evaluated based on one’s standards (Shin and Johnson, 1978; Pavot and Diener, 1993). As a key indicator of SWB, LS life satisfaction focuses on identifying and using strengths as buffers against the development of psychopathological problems (Veenhoven, 1984; Proctor et al., 2009).

Similar to adults, life satisfaction appears to be relatively stable as most adolescents generally report a positive attitude toward life (Gilman and Huebner, 2003; Moksnes and Espnes, 2013; Opshaugh, 2013). Euro-American studies have found little differences between males and females. When small gender differences were found, boys usually reported higher LS (Gilman and Huebner, 2003). Culturally, the differences between countries are not substantial, only high individualism (vs. collectivism) consistently predicted differences in LS between nations when controlling for other variables (Diener et al., 1995). Based on findings from the early 1990s, Czech adolescents did not differ significantly in LS from adolescents across Europe (Macek, 1999). Nevertheless, some studies have shown that adolescents and adults in Eastern European countries scored slightly lower on LS compared to Western Europeans (Delhey, 2004).

### Daily hassles

Daily hassles (DHs) represent “experiences and conditions of daily living that have been appraised as salient and harmful or threatening to the endorser’s well-being” (Lazarus, 1986, p. 40). These recurring events, being a part of the micro-social environment of adolescents’ lives, contribute to adolescents’ negative feelings and irritability (DeLongis et al., 1982; Kanner et al., 1987). As chronic stressors, they affect SWB and mental health (Asselmann et al., 2017). DHs have a cumulative effect (Kanner et al., 1987); when they affect multiple domains of adolescents’ lives with high frequency, stress levels increase significantly. Difficulties in specific domains of life (school, family, and peers) may be of varying importance to adolescents (Gelhaar et al., 2007; Booth and Anthony, 2015; Mize and Kliewer, 2017).

Most frequently reported problems and stressors come from the school context, family relationships, problems associated with risk behavior, health problems, and problems with romantic partners. Recently, problems with finances, leisure time, privacy and online activities, and personal psychological problems have also become increasingly common (Cheng and Li, 2010; Mize and Kliewer, 2017; Zorbaz et al., 2020).

Several studies have reported the specific effect of DHs on SWB and especially on life satisfaction (Huebner, 1991; Proctor et al., 2009; Cheng and Li, 2010; Udayar et al., 2021). For example, minor daily events and everyday difficulties (e.g., arguments with friends, poor exam performance, and enjoyment of a hobby) have been shown to contribute to unique variance over and above major stressful life events (Gilman and Huebner, 2003; Proctor et al., 2009).

### Self-esteem

Self-esteem as a global self-evaluation fits well together with life satisfaction considerations of adolescents’ SBW (Diener, 1984; Grob et al., 1996; Harter, 2006; Moksnes and Espnes, 2013). Diener and Diener (2009) found that self-esteem is a powerful predictor of life satisfaction, especially in individualist cultures (Kwan et al., 1997). Studies have shown that in adulthood, self-esteem is a relatively stable personality characteristic that, in relation to life satisfaction, can explain between-person variation (Anusic and Schimmack, 2016). However, in early and middle adolescence, self-esteem is still forming and is more dependent on situational influences and interpersonal evaluations (Kuster and Orth, 2013; Sánchez-Queija et al., 2017).

The association between self-esteem and different types of personal difficulties has been documented theoretically and empirically. A social-cognitive explanation of depression (Oatley and Bolton, 1985; Higgins, 1987; Kernis et al., 1998) assumes that depriving one’s self-defining role and deflating self-esteem are related to how one perceives and handles difficulties and problem situations (Cheng and Lam, 1997; Mann et al., 2004). Self-esteem is usually positively correlated with life satisfaction (Moksnes and Espnes, 2013), but the specific role of DHs in this association is unclear. Therefore, we propose a model in our study where we consider self-esteem and daily hassles as predictors of perceived life satisfaction.

### Adolescent life in the changing Czech society

We compared Czech adolescents from different times, so a brief description of the social changes in this period is necessary for a better understanding of the macrosocial influences on the everyday life of adolescents.

The transformation to a democratic political system after the fall of the communist regime at the end of 1989 brought many economic and social changes in Czech society. Adolescents and their parents were experiencing a kind of “social moratorium.” They reassessed their lives and sought a new personal perspective, seeing their future optimistically but rather vaguely. In the late 1990s, the social situation changed dramatically, as economic, political and legislative problems led to many Czechs experiencing disillusionment with the social situation, and their optimism decreased considerably (Macek and Marková, 2004; Linek, 2010).

The 2000’s could be metaphorically described as “entering adulthood” of the Czech society. Unrealistic optimism and naive confidence in the future were replaced by a more realistic view of the future (Linek, 2010). Adolescents raised in a democratic society take individual freedom for granted; and their possibilities for self-development were greatly expanded. However, the pressure for independent decision-making and the associated uncertainty have also increased. These adolescents are more individualistic than before, recognizing the value of education, success, social prestige, leisure, and entertainment. They are very tolerant and liberal in relation to drugs and sex, less dependent on parents and teachers (Macek et al., 2013).

All these characteristics can apply to the life of adolescents in the 2010s. A new element has been the massive expansion of social networking, still mainly through personal computers, laptops, and tablets. Thus, life on social networking sites was still separate from other activities of adolescents (Subrahmanyam and Šmahel, 2011). The globalization of adolescent culture and socialization through media has progressed quickly.

The last cohort, born in the new millennium, grew up with mobile smartphones becoming the main means of communication and the platform for adolescents’ long-term and often permanent participation in social networks. Twenge (2017) called them the iGeneration of adolescents, for whom social media and communication through text and picture messaging have partially replaced normal face-to-face social interactions and other activities. They spend less time with peers than previous generations, and despite spending more time at home, often in the presence of parents, their communication with parents is limited. In the virtual social world, they face unrealistic expectations, aggressiveness, and inadequate benchmarks for their self-assessment. This may be why they are more likely to experience anxiety, depressive symptoms, and loneliness. They are very tolerant but impatient and indecisive (Twenge, 2017). However, as prior research showed, the effects of technology use and new modes of communication on adolescents’ well-being can be both positive and negative because of a complex interplay of different factors (Bedrošová et al., 2018; Dedkova et al., 2022).

### The present study

This study offers a unique opportunity to analyze well-being data from four cohorts (1992, 2001, 2011, and 2019). The primary goal was to describe the distribution of self-reported life satisfaction and daily hassles across saliently different macrosocial situations. Based on previous research, we assumed life satisfaction should remain stable. The daily hassles are more likely to be different now than in the past because they reflect the different macrosocial contexts.

The second goal of the study is to assess whether self-reported daily hassles are related to self-reported life satisfaction and whether this relationship differs across cohorts. Since self-esteem affects the assessment of life satisfaction, we assessed this relationship separately while controlling for self-esteem.

## Methods

### Participants and procedure

Four cohorts of Czech adolescents were sampled about 10 years apart in 1992, 2001, 2011, and 2019. All are quota samples reflecting the population of the Czech Republic with respect to the type of lower and higher secondary schools attended; whole classrooms were sampled. The samples covered two grade cohorts, specifically, grade 8 students around age 14 (age 13, *p* = 4.3%, 14, *p* = 22.4%, 15, *p* = 22.4%) and grade 10 students around age 16 (age 16, *p* = 27.4%, 17, *p* = 16.1%, over 17, *p* = 7.3%).

The 1992 sample was collected in the Euronet Pilot Study (Alsaker and Flammer, 1999; *N* = 257, 44% female). The participants directly experienced the socialist regime and the start of the transformation in 1989. The 2001 and subsequent data were collected to replicate the 1992 study. Participants in the 2001 sample (*N* = 310, 51% female) grew up in the transformation turmoil following the 1989 revolution. The 2011 sample (*N* = 371, 47% female) was computer-administered in classrooms. Participants in this sample were born during the transformation period, grew up in a consolidated EU-member country, and had Internet services as a part of their everyday life. The 2019 sample (*N* = 3,206, 56% female) was mostly administered in classrooms using pen-and-paper format (2,484, 77.5%), while some were administered on school computers (722, 22.5%). This generation grew up in a society increasingly influenced by online media and social networks.

The total effective sample size was *N* = 4,005 (*n*_1992_ = 255, *n*_2001_ = 305, *n*_2011_ = 363, and *n*_2019_ = 3,082).

The informed consent procedures reflected the contemporary local standards. In 1992 and 2001, schools used their authority to administer the questionnaire as a part of curriculum. Parental informed consent was sought in 2011 and 2019. The 2019 survey was approved by the Research Ethics Committee of Masaryk University (Ref. No. EKV-2018-026).

### Measures

*Well-being*A total of 13 items addressing adolescents’ subjective well-being were selected from the Berne Questionnaire on Adolescents’ Well-Being (BSW-Y; Grob et al., 1991). They represent two factors, namely life satisfaction and self-esteem. The BSW-Y has been shown to have satisfactory psychometric properties (Grob et al., 1991; Grob, 1995). The items use a 4-point response scale from 1 (totally false) to 4 (very true). The 8-items of *life satisfaction* (LS) scale fit a unidimensional model (ordinal CFA robust CFI = 0.967, SRMR = 0.043), with all items loading well on the factor of life satisfaction, resulting in sufficient internal consistency (McDonald’s omega = 0.75). The 5-item scale of self-esteem also fit a simple unidimensional model (ordinal CFA robust CFI = 0.945, SRMR = 0.056), with all items loading well on the single factor, resulting in sufficient internal consistency (McDonald’s omega = 0.75).

*Daily hassles (DH)*The inventory developed in the Euronet Pilot Study (Alsaker et al., 1999) uses 11 items asking about the extent to which selected life domains (for the list see Table 2) presented obstacles or difficulties to the respondent over the previous 6 months. The items use a 4-point response scale from 1 (not at all difficult) to 4 (very difficult). Responses were summed to indicate the overall level of perceived daily hassles. This sum is not intended as a reflective measurement of a construct but as a formative index of the total amount of perceived hassles in the 11 domains. The polychoric correlations among items range from 0.05 to 0.39 suggesting (1) no strong latent variable is producing the perceived hassles (2) there are no compensatory relationships between the domains of hassles in which perceiving problems in one domain would prevent or reduce perceiving hassles in another. With only cross-sectional data we can estimate reliability with McDonald’s omega total = 0.77 estimating the proportion of all systematic variance in an unweighted composite score (Revelle and Condon, 2019).

**Table 1:** Descriptive statistic of life satisfaction, self-esteem, and total daily hassles scores by cohort.

	*N*	*M*	*SD*	Minimum (1)	Maximum (4)	Skewness	Kurtosis
Life satisfaction							
1992	254	2.87	0.42	1.38	3.88	−0.43	0.63
2001	305	2.95	0.43	1.63	4.00	−0.34	0.09
2011	362	2.92	0.49	1.38	4.00	−0.57	0.34
2019	3,063	2.98	0.46	1.00	4.00	−0.64	0.48
Self-esteem							
1992	255	2.87	0.50	1.40	4.00	−0.17	−0.35
2001	303	2.92	0.53	1.40	4.00	−0.35	−0.11
2011	362	2.90	0.59	1.20	4.00	−0.31	−0.41
2019	3,054	2.77	0.62	1.00	4.00	−0.39	−0.30
Daily hassles total							
1992	255	1.90	0.37	1.00	2.91	0.35	−0.19
2001	301	1.93	0.35	1.00	3.18	0.28	−0.09
2011	363	2.02	0.44	1.00	4.00	0.79	1.87
2019	3,062	1.90	0.44	1.00	4.00	0.50	0.29

**Table 2:** Descriptive statistics of individual daily hassles domains by cohort.

	Cohort									
	1992		2001		2011		2019			
	M	SD	M	SD	M	SD	M	SD	Welch *p*	*ω* ^2^
School	2.64	0.82	2.63	0.70	2.75	0.85	2.60	0.85	0.010	0.002
Money	2.36	0.91	2.36	0.89	2.33	0.89	1.91	0.83	<0.001	0.045
Boy/girlfriend	2.10	0.98	2.18	1.03	2.29	1.11	2.09	1.15	0.009	0.002
Friends & peers	1.57	0.72	1.54	0.66	1.79	0.85	1.93	0.89	<0.001	0.022
Active sports	1.78	0.92	1.92	0.99	2.01	0.99	1.99	0.97	0.004	0.003
Parents and family	1.91	0.91	2.05	0.89	2.20	1.03	1.98	0.97	<0.001	0.004
Home and neighbors	1.54	0.80	1.54	0.81	1.63	0.88	1.46	0.76	0.002	0.004
Health	1.73	0.84	1.74	0.78	1.84	0.82	1.81	0.87	0.179	<0.001
Leisure time	1.67	0.88	1.66	0.82	1.69	0.84	1.77	0.88	0.024	0.002
Public information access	1.77	0.74	1.85	0.81	1.83	0.78	1.71	0.73	0.002	0.003
Own room	1.82	1.07	1.80	0.97	1.88	1.05	1.64	0.92	<0.001	0.007

The theoretical range was 1–4. Higher values represent a higher perceived level of hassles. Welch *p* is the *p*-value of the F-test with 3 and 570–614 degrees of freedom with Welch’s correction.

### Analysis

Linear models were estimated in IBM SPSS 28 using the GENLIN procedure with robust standard errors. Models with individual hassles were run on 10 multiple-imputed datasets to overcome the missing responses in hassles with boy/girlfriend. The pooled values of parameters reported below did not meaningfully differ from the parameters found in the unimputed data.

Validity checks on the well-being scale and hassles items included number of valid responses, too fast responses, and strings of identical responses. This excluded 2, 5, 8, and 124 respondents from the respective cohorts.

Measurement models were estimated in R (R Core Team, 2021) with the lavaan package (Rosseel, 2012) and omega total was estimated with package psych (Revelle, 2022).

## Results

### Descriptive statistics

Life satisfaction scores were slightly negatively skewed across the four cohorts. Its means and standard deviations (Table 1) changed very little over time [Welch’s *F*(3, 598.4) = 5.99, *p* < 0.001, *ω*^2^ = 0.003]. The gender effect was small, with males reporting slightly higher life satisfaction [Welch’s *F*(1, 3,913) = 34.2, *p* < 0.001, *ω*^2^ = 0.008].

Self-esteem showed similar distribution with minimal cohort differences [Welch’s *F*(3, 607.0) = 11.9, *p* < 0.001, *ω*^2^ = 0.007]. The gender effect was small, with males reporting slightly higher self-esteem [Welch’s *F*(1, 3,862) = 182.1, *p* < 0.001, *ω*^2^ = 0.044]. Life satisfaction and self-esteem were positively correlated across cohorts (*r*’s = 0.48, 0.56, 0.61, and 0.63, respectively).

Descriptive statistics of individual daily hassles treated as a continuous variable are presented in Table 2 separately by cohort. The means were all below 3, corresponding to “somewhat bothersome,” although most values were below 2, indicating “not very bothersome.” Table 1 reports the descriptive statistics of the total daily hassles (the mean of individual hassles). The cohort differences in means of DH were minimal [Welch’s *F*(3, 603.9) = 8.9, *p* < 0.001, *ω*^2^ = 0.006]. Gender differences in mean DH differed across cohorts. In 2019, females reported higher level of DH (Cohen *d* = 0.40), whereas the difference between earlier cohorts were close to zero [interaction *F*(3, 707.1) = 12.8, *p* < 0.001, *ω*^2^ = 0.009].

### Linear models predicting life satisfaction

The primary aim of the analysis was to see whether self-reported daily hassles in life are related to self-reported LS and whether this relationship differs across cohorts. We used standardized LS as the outcome in a linear model and gender (the male was the reference category), cohort (2019 was the reference category), and standardized self-esteem as predictors. In separate models, DH entered either as individual predictors or as a single variable representing the overall level of hassles.

The models reported in Table 3 included interactions between gender and DH, cohort and DH, and cohort and gender. We also estimated a model with a three-way interaction between gender, cohort, and DH, but this interaction did not contribute to the explained variance while using many degrees of freedom.

**Table 3:** Parameters of the linear models predicting life satisfaction.

Parameter	M1a	M1b	M2a	M2b
	*B* (*SE*)	*B* (*SE*)	*B* (*SE*)	*B* (*SE*)
(Intercept)	0.97 (0.06)	0.39 (0.05)	0.93 (0.05)	0.36 (0.05)
Cohort				
1992	−0.20 (0.17)	−0.09 (0.15)	**−0.38 (0.15)**	**−0.28 (0.13)**
2001	−0.08 (0.19)	−0.04 (0.16)	0.05 (0.16)	0.07 (0.13)
2011	0.05 (0.16)	0.06 (0.13)	−0.05 (0.14)	−0.04 (0.13)
Gender = female	0.12 (0.08)	**0.22 (0.06)**	0.14 (0.07)	0.25 (0.06)
Cohort X gender = female				
1992	**−0.34 (0.12)**	−0.26 (0.11)	**−0.40 (0.12)**	**−0.24 (0.10)**
2001	−0.13 (0.11)	−0.16 (0.09)	−0.13 (0.11)	−0.13 (0.09)
2011	−0.26 (0.11)	**−0.24 (0.09)**	−0.30 (0.11)	−0.22 (0.09)
Self-esteem (standardized)		**0.53 (0.02)**		
*Daily hassles* (semi-standardized)			**−0.43 (0.03)**	**−0.18 (0.02)**
School	**−0.09 (0.03)**	−0.06 (0.03)		
Money	**−0.15 (0.03)**	**−0.12 (0.03)**		
Boy/girlfriend	**−0.10 (0.02)**	−0.02 (0.02)		
Friends and peers	**−0.09 (0.03)**	0.06 (0.03)		
Active sports	−0.06 (0.02)	−0.03 (0.02)		
Parents and family	**−0.14 (0.03)**	**−0.07 (0.03)**		
Home and neighbors	−0.01 (0.03)	0.01 (0.03)		
Health	**−0.09 (0.03)**	−0.05 (0.03)		
Leisure time	**−0.10 (0.03)**	−0.05 (0.03)		
Public information access	**−0.14 (0.03)**	**−0.10 (0.03)**		
Own room	−0.02 (0.03)	−0.01 (0.03)		
*Cohort X daily hassles*			0.16 (0.06)	0.04 (0.06)
1992				
2001			−0.01 (0.07)	−0.08 (0.05)
2011			0.08 (0.06)	−0.01 (0.05)
School * 1992	**−0.19 (0.07)**	**−0.20 (0.06)**		
2001	0.10 (0.09)	0.08 (0.07)		
2011	−0.14 (0.06)	−0.11 (0.05)		
Money * 1992	0.13 (0.07)	0.08 (0.06)		
2001	0.02 (0.06)	−0.02 (0.05)		
2011	0.05 (0.07)	0.05 (0.06)		
Boy/girlfriend * 1992	0.08 (0.06)	0.07 (0.06)		
2001	−0.01 (0.06)	−0.02 (0.05)		
2011	−0.04 (0.05)	−0.03 (0.04)		
Friends and peers * 1992	−0.03 (0.10)	−0.06 (0.08)		
2001	0.03 (0.08)	0.05 (0.07)		
2011	0.03 (0.07)	0.03 (0.06)		
Active sports * 1992	0.04 (0.06)	0.00 (0.06)		
2001	−0.05 (0.06)	−0.06 (0.05)		
2011	0.07 (0.06)	0.03 (0.05)		
Parents and family * 1992	0.01 (0.07)	0.01 (0.06)		
2001	0.04 (0.06)	−0.03 (0.05)		
2011	−0.06 (0.06)	−0.07 (0.05)		
Home and neighbors * 1992	0.02 (0.06)	−0.05 (0.06)		
2001	0.00 (0.07)	0.03 (0.06)		
2011	0.02 (0.06)	−0.04 (0.05)		
Health * 1992	0.05 (0.07)	0.03 (0.06)		
2001	0.02 (0.08)	0.07 (0.06)		
2011	0.08 (0.07)	0.08 (0.07)		
Leisure time * 1992	0.01 (0.07)	−0.01 (0.07)		
2001	−0.09 (0.07)	−0.09 (0.06)		
2011	0.06 (0.08)	0.03 (0.07)		
Public information access * 1992	0.13 (0.09)	0.19 (0.08)		
2001	0.01 (0.07)	0.04 (0.06)		
2011	**0.20 (0.07)**	0.05 (0.06)		
Own room * 1992	0.02 (0.06)	−0.01 (0.05)		
2001	−0.02 (0.06)	−0.09 (0.05)		
2011	0.00 (0.06)	0.01 (0.05)		
Gender = female X daily hassles			−0.07 (0.03)	−0.06 (0.03)
Female * school	−0.02 (0.04)	0.00 (0.03)		
Female * money	0.05 (0.04)	0.08 (0.03)		
Female * boy/girlfriend	0.00 (0.03)	−0.03 (0.02)		
Female * friends and peers	0.03 (0.04)	−0.04 (0.03)		
Female * active sports	0.02 (0.03)	0.00 (0.03)		
Female * parents and family	**−0.13 (0.04)**	**−0.10 (0.03)**		
Female * home and neighbors	−0.09 (0.04)	−0.08 (0.03)		
Female * health	0.01 (0.04)	0.02 (0.04)		
Female * leisure time	0.00 (0.04)	0.00 (0.03)		
Female * public information access	0.00 (0.04)	0.05 (0.04)		
Female * own room	−0.02 (0.04)	0.00 (0.03)		
*R* ^2^	0.239	0.440	0.209	0.417

Effects in bold, *p* < 0.01. Reference categories are 2019 and male. Life satisfaction and self-esteem in the model are standardized—B’s can be interpreted as betas or Cohen *d*’s. Daily hassles were recoded to a scale from 0 (not at all difficult) to 3 (very difficult) and the overall daily hassles score has been divided by its standard deviation so that it expresses the amount of hassles as a multiple of SDs from zero hassles.

*The model with individual daily hassles* (M1a and M1b in Table 3). The cohorts did not differ significantly in life satisfaction when DH were zero [Wald *χ*^2^(3) = 4.97, *p* = 0.174]. Gender differences in LS were small, ranging from Cohen *d* of 0.22 in favor of males in 1992 to *d* = 0.12 in favor of females in 2019; still, gender difference across cohorts was significant [Wald *χ*^2^(3) = 12.8, *p* = 0.005].

The effect of DH was in the expected direction. For most categories of hassles, their unique effects were small but statistically significant. The difference in mean LS between no reported hassles (coded 0) and “very difficult” hassles (coded 3) was around 0.3 standard deviation of LS in the latest (reference) cohort. The categories with the greatest effect were hassles related to money, parents and family, and access to public information. On the other hand, hassles related to sports, neighbors, or having their own room did not have a unique effect on LS. The unique effects of hassles did not differ much between cohorts. Only hassles related to school appeared to have a more pronounced effect in 1992 and 2011.

Conversely, hassles related to money had a much smaller effect in 1992. In 1992 and 2011, the effect of hassles related to public information access was much smaller. Apart from hassles related to parents and family, where the effect was almost double for females, and to a lesser extent hassles related to neighbors, the hassles effects were independent of gender.

Adding self-esteem to the model decreased most of the effects due to collinearity, as expected. The effect of self-esteem was understandably large (*B* = 0.53, *p* < 0.001). The inclusion of standardized self-esteem as another predictor changed the effects, as they now represented the unique effects of hassles on the part of LS variance not affected by or related to self-esteem. The effects of hassles related to money and parents/family were the least affected by the inclusion of self-esteem and remained statistically significant. The effect of hassles related to public information access was independent of self-esteem. The effect of hassles related to parents and family was still much higher in females.

*The model with total daily hassles* (M2a and M2b in Table 3). The effect of total DH on LS was medium and significant (*B* = −0.43, *p* < 0.001). In 1992, the effect was smaller than in the later cohorts. When self-esteem was controlled, the effect of total DH decreased to less than half but remained significant. The effect of DH was slightly but non-significantly higher in females than in males.

Here, the interaction between gender and cohort was clearer. In all the earlier cohorts, gender differences in mean life satisfaction were minimal, whereas, in the 2019 cohort, females reported higher satisfaction than males by about a third of standard deviation, especially when controlling for self-esteem.

## Discussion

The present study aimed to describe changes in life satisfaction of Czech adolescents over the past three decades of social change. Our general assumption was that social processes and changes in Czech society affect adolescents’ everyday lives and lived experiences and consequently life satisfaction, which was the focus of our analyses. Although we could not assess the direct influence of social changes, we can put our findings in the context of other studies that investigated life satisfaction around the time of our study.

According to the European Values Study, Czech adults experienced an increase in LS in the first decade following the regime change (1990–1999). Since 2008, no significant increase in LS has been observed (Večerník and Mysíková, 2014). A similar result was found in the HBSC study, which tracked the LS of Czech adolescents from 2002 to 2014. The adolescents in our study seemed to follow this general trend. The raw means of LS did not differ much across cohorts, but when we accounted for gender and DHs, life satisfaction in the 1992 cohort was much lower than in the later cohorts. Since this effect differed when we included individual hassles in the model, the cohort differences in the structure DHs likely obscured cohort differences in raw life satisfaction.

Regarding gender differences, boys usually report as slightly more satisfied than girls (Hodačová et al., 2017). When accounting for hassles, the females reported higher life satisfaction, except for 1992, when males scored higher on life satisfaction. Hence, the male life satisfaction advantage appears to be due to fewer perceived hassles. We found support for the assumption that self-esteem is a stable predictor of adolescents’ LS (e.g., Diener and Diener, 2009; Moksnes and Espnes, 2013). We observed minimal gender differences, except in 2019 when girls reported higher life satisfaction after controlling for self-esteem, which is consistent with Zeigler-Hill and Myers (2012). Self-esteem is usually more stable compared to life satisfaction (Diener and Diener, 2009; Anusic and Schimmack, 2016). Our results are consistent with these findings, with the four cohorts of adolescents differing only minimally in the self-esteem means.

The perceived volume of daily hassles was found to be highly predictive of LS. Even when controlling for demographic variables, LS quickly decreased with daily hassles. The association appeared stable over time, although the link was weaker in 1992. This finding can be interpreted based on a macrosocial view of adolescents’ lives: the euphoria after the fall of communism and new opportunities led to an unusually high level of life optimism among most young Czechs in the first half of the 1990s. Everyday difficulties were more often viewed as necessary but only temporary consequences of the rapid social change moderating their negative effect on life satisfaction. In the following years, economic conditions declined along with the level of optimism in the Czech population (Linek, 2010). Czechs became more skeptical of their life expectations, and their life satisfaction was more linked to their everyday experience. Therefore, the link between perceptions of daily hassles and life satisfaction was stronger for other cohorts as well.

While all the domains of daily hassles contribute to the decrease in LS, their unique effects differ. Problems experienced by adolescents at school were the biggest contributors to decreased LS, because they were also the most frequent domain of hassles (*cf.* Barrett and Heubeck, 2000; Booth and Anthony, 2015). School difficulties differed from other difficulties in that they had a higher effect on LS in 1992. Schools’ social influence in the communist era was still high in 1992, leading to the school difficulties being experienced as more serious. Although fears related to school are the most frequent in Czech adolescents (Michalčáková et al., 2013), we found that influence of daily hassles related to the school on life satisfaction slightly declined later. It may be related to the fact that students could in this period perceive problems at school as less severe or important in the overall context of their life in which adolescents started to be newly partially influenced by social media (Twenge, 2019).

Problems with money were very frequent across cohorts except the last one. For adolescents, this is primarily a parental support issue (Lavee and Ben-Ari, 2008; Suarez-Morales and Lopez, 2009) so the significant reduction in the incidence of money problems in the last cohort may be due to the recent increase in the standard of living of Czech families (Kuchařová et al., 2019).

All our findings must be considered in light of the fact that there were uncontrolled sampling differences between the individual years. Although the sampling procedure was the same, schools’ motivations to participate, particularly parents’ willingness to provide informed consent, have changed over the three decades. In 1992, the schools exercised the authority to administer surveys as a part of the curriculum without explicit consent from parents. This has gradually changed, and in 2011 and 2019, individual parental consent needed to be secured, with considerable proportions of students not participating in the survey.

The list of daily hassle domains has not changed since the first cohort in 1992. The relevance of individual domains of hassles may have changed and new relevant domains may have emerged. This presents a challenge for the formative index of the total daily hassles because formative indicators are not interchangeable and because the assumption of the constancy of (unit) weights may not hold. That questions the validity of the model using total hassles. However, the model with individual hassles shows that apart from school-related and public-information-access-related hassles the magnitude of association with life satisfaction does not change significantly.

Despite its limitations, our study sends a positive message about the well-being of Czech adolescents. The data show that their subjective well-being, both in the form of life satisfaction and self-esteem, has been stable over the past three decades despite the macrosocial changes the Czech society has undergone over the period. Although the frequency of daily problems has changed slightly over these years, their impact on their life satisfaction has not changed much.

## Data availability statement

The raw data supporting the conclusions of this article will be made available by the authors, without undue reservation.

## Ethics statement

The 2019 research project was approved by the Research Ethics Committee of Masaryk University (Ref. No. EKV-2018-026). Written informed consent to participate in this study was provided by the participants’ legal guardian/next of kin.

## Author contributions

PM and SJ contributed to the conception and design of the study. LL organized the database. SJ performed the statistical analysis. PM wrote the first draft of the manuscript. PM, SJ, and LL wrote sections of the manuscript. All authors contributed to the article and approved the submitted version.

## Funding

This work was supported by the Czech Science Foundation (grant number GA19-22997S) and also supported by Masaryk University, Faculty of Social Studies.

## Conflict of interest

The authors declare that the research was conducted in the absence of any commercial or financial relationships that could be construed as a potential conflict of interest.

## Publisher’s note

All claims expressed in this article are solely those of the authors and do not necessarily represent those of their affiliated organizations, or those of the publisher, the editors and the reviewers. Any product that may be evaluated in this article, or claim that may be made by its manufacturer, is not guaranteed or endorsed by the publisher.
